# Antitumor effects of a novel photosensitizer-mediated photodynamic therapy and its influence on the cell transcriptome

**DOI:** 10.32604/or.2023.042384

**Published:** 2024-04-23

**Authors:** JINGJING CHEN, DAN WANG, ZEQUN WANG, MENGYUAN HAN, HOUQING YIN, WENTING ZHOU, RIBAI YAN, YAN PAN

**Affiliations:** 1 Department of Pharmacology, School of Pharmacy, Changzhi Medical College, Changzhi, China; 2 Department of Pharmacology, School of Basic Medical Sciences, Health Science Center, Peking University, Beijing, China; 3 Beijing Key Laboratory of Tumor Systems Biology, Peking University, Beijing, China; 4 Department of Pharmacology, Xinjiang Medical University, Urumqi, China; 5 School of Pharmaceutical Sciences, Peking University, Beijing, China

**Keywords:** Photodynamic therapy, ROS, Apoptosis, Autophagy, Bioinformatic analysis

## Abstract

Photodynamic therapy (PDT) is a promising cancer treatment. This study investigated the antitumor effects and mechanisms of a novel photosensitizer meso-5-[ρ-diethylene triamine pentaacetic acid-aminophenyl]−10,15,20-triphenyl-porphyrin (DTP) mediated PDT (DTP-PDT). Cell viability, reactive oxygen species (ROS), and apoptosis were measured with a Cell Counting Kit-8 assay, DCFH-DA fluorescent probe, and Hoechst staining, respectively. Cell apoptosis- and autophagy-related proteins were examined using western blotting. RNA sequencing was used to screen differentially expressed mRNAs (DERs), and bioinformatic analysis was performed to identify the major biological events after DTP-PDT. Our results show that DTP-PDT inhibited cell growth and induced ROS generation in MCF-7 and SGC7901 cells. The ROS scavenger N-acetyl-L-cysteine (NAC) and the P38 MAPK inhibitor SB203580 alleviated DTP-PDT-induced cytotoxicity. DTP-PDT induced cell apoptosis together with upregulated Bax and downregulated Bcl-2, which could also be inhibited by NAC or SB203580. The level of LC3B-II, a marker of autophagy, was increased by DTP-PDT. A total of 3496 DERs were obtained after DTP-PDT. Gene Ontology and Kyoto Encyclopedia of Genes and Genomes analyses indicated that DERs included those involved in cytosolic ribosomes, the nuclear lumen, protein binding, cell cycle, protein targeting to the endoplasmic reticulum, and ribosomal DNA replication. Disease Ontology and Reactome enrichment analyses indicated that DERs were associated with a variety of cancers and cell cycle checkpoints. Protein-protein interaction results demonstrated that *cdk1* and *rps27a* ranked in the top 10 interacting genes. Therefore, DTP-PDT could inhibit cell growth and induce cell apoptosis and autophagy, partly through ROS and the P38 MAPK signaling pathway. Genes associated with the cell cycle, ribosomes, DNA replication, and protein binding may be the key changes in DTP-PDT-mediated cytotoxicity.

## Introduction

Cancer is a major public health problem and the leading cause of death worldwide. In 2020, there were 19.3 million new cases of cancer and 10 million deaths [1]. Traditional and new treatments, including surgery, radiation, chemotherapy, targeted therapy, and immunotherapy, have produced some therapeutic benefits but often have severe side effects or are susceptible to drug resistance, which limits their clinical application [2,3]. Approaches to improve the therapeutic effect and reduce the adverse reactions of cancer treatment include enhancing the drug stability and drug loading capacity of drug delivery systems, preparing nanoparticles and polymer prodrugs, and developing drugs using new mechanisms, such as cuproptosis [4–7].

Photodynamic therapy (PDT) is a promising anticancer approach that is based on the application of a nontoxic photosensitizer that accumulates selectively in cancer tissues. When irradiated by laser-emitted light of an appropriate wavelength, the photosensitizer is converted from the singlet basic energy state S° into the excited singlet state S^1^, which exists for a short time and quickly transforms into the excited triplet state T^1^ with a longer lifespan. Then, the excited triplet state T^1^ releases energy back to the ground state, during which the released energy is transferred to molecular oxygen, leading to the formation of reactive oxygen species (ROS) [8]. These ROS can oxidize cellular macromolecules, including lipids, proteins, and nucleic acids, resulting in cell death [8]. Compared with conventional anticancer therapy, PDT has several advantages, including less invasiveness, precise tumor targeting, minimal systemic toxicity, and the availability of repeated treatments [9]. Currently, PDT has been approved for the clinical treatment of a variety of tumours [10].

RNA sequencing is the most commonly used gene detection technology and can effectively perform genome-wide relative quantification of gene expression on the basis of comprehensive and rapid access to the set of all transcripts in a specific cell or tissue of a species. RNA sequencing is widely used in transcriptomic studies, and the detection of differentially expressed mRNAs (DERs) was performed. The screening and biological annotation of DERs have been the most commonly used method in recent years to explore the differences in biological functions after drug treatment [11].

**Figure 1:**
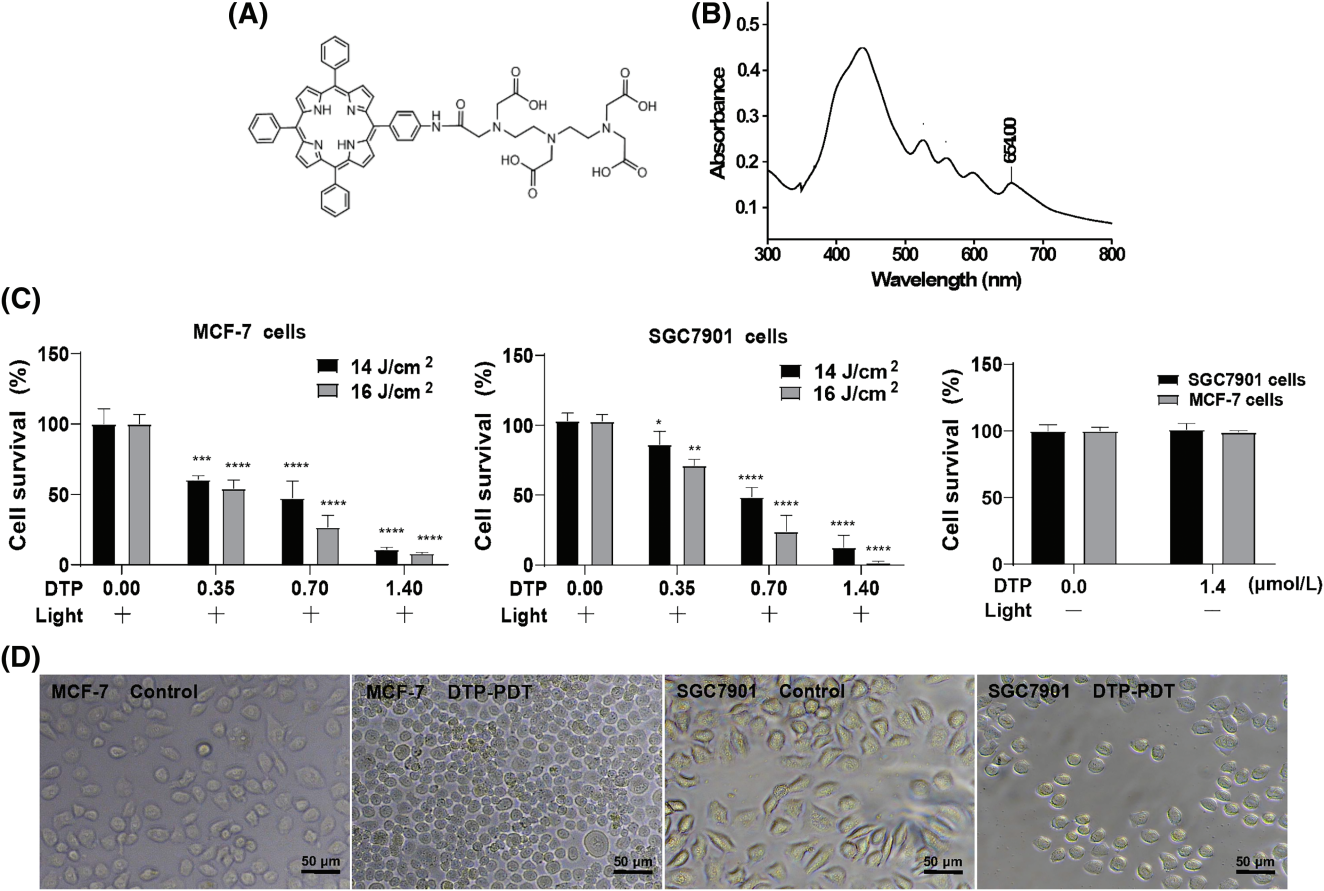
Inhibitory effects of DTP-mediated photodynamic therapy on MCF-7 and SGC7901 cell lines. (A) Chemical structure of DTP. (B) The UV‒Vis absorption spectrum of DTP. Cell viability analysis of (C) MCF-7 cells and SGC7901 cells with different concentrations of DTP (0, 0.35, 0.7, and 1.4 µM) and energy densities of light illumination (14 or 16 J/cm^2^) at a wavelength of 650 nm. Eight hours after irradiation, cell viability was tested using CCK-8 assay. *p < 0.05 *vs*. the control group; **p < 0.01 *vs*. the control group; ***p < 0.001 *vs*. the control group; and ****p < 0.0001 *vs*. the control group. Cell viability analysis of MCF-7 and SGC7901 cells with 1.4 µM DTP without light illumination. (D) Morphological changes in MCF-7 (left two pictures) and SGC7901 (right two pictures) cells exposed to DTP-PDT. Scale bar = 50 μm.

Meso-5-[ρ-diethylene triamine pentaacetic acid-aminophenyl]−10,15,20-triphenyl-porphyrin (DTP) is a novel porphyrin-based photosensitizer and has a specific absorption peak at 650 nm. Its chemical structure is displayed in Fig. 1A. Here, we investigate the effects of DTP-mediated PDT (DTP-PDT) on the human breast cancer cell line MCF-7 and the human gastric cancer cell line SGC7901 using RNA sequencing combined with bioinformatic analysis.

## Materials and Methods

### Preparation of DTP

The molecular formula of DTP is C_58_H_52_N_8_O_9_ (Fig. 1A), and its molecular weight is 1004.39. The detailed synthetic process of DTP is demonstrated in our patent (CN 201010135433.X), and the general synthetic process is described in reference [12].

### Cell culture

The human breast cancer cell line MCF-7 was donated by Liu Zhaofei’s lab (Peking University, China), and the human gastric cancer cell line SGC7901 was obtained from Beijing Cancer Hospital, China. Cells were incubated in Dulbecco’s modified Eagle’s medium supplemented with 10% fetal bovine serum (Gemini, Uruguay), 100 IU/mL penicillin, and 100 µg/mL streptomycin at 37°C in a 5% CO_2_ incubator.

### DTP-mediated photodynamic therapy

MCF-7 and SGC7901 cells were seeded into 96-well plates at a density of 5 × 10^3^ cells/well for 24 h. The cells were incubated with different concentrations of DTP (0, 0.35, 0.7, and 1.4 μM) for 24 h followed by photoirradiation with an energy density of 14 J/cm^2^ for 14 min or 16 J/cm^2^ for 17 min at a wavelength of 650 nm. The cells were also incubated in the presence of 12 μM P38 inhibitor SB203580 (Beyotime, China), 12 μM JNK inhibitor SP600125 (Beyotime, China), 12 μM ERK inhibitor FR180204 (Beyotime), or 10 mM ROS inhibitor N-acetyl-L-cysteine (NAC; Beyotime). Each experimental group included four wells per group. Eight hours later, the cells were treated with 10 µL of Cell Counting Kit-8 (CCK-8; Beyotime) for 2 h at 37°C. Absorbance was measured at 450 nm using a microplate reader. The cell survival rate was calculated using the following formula: Cell survival rate (%) = ([OD_experimental_ − OD_blank_]/[OD_control_ − OD_blank_]) × 100.

### Measurement of intracellular ROS

The generation of intracellular ROS was detected using the oxidant-sensitive fluorescent probe 2,7-dichlorofluorescein diacetate (DCFH-DA; Sigma, USA). MCF-7 and SGC7901 cells were seeded into 24-well plates overnight and then incubated with different concentrations of DTP for 24 h followed by light illumination at 16 J/cm^2^ for 17 min. Three hours later, the cells were incubated with 10 µM DCFH-DA for 15 min at 37°C in the dark. After rinsing with phosphate-buffered saline (PBS) three times, the cells were visualized under a fluorescence microscope. Hydrogen peroxide (30 μM; Applygen, China) was used as the positive control.

### Hoechst 33342 staining

MCF-7 and SGC7901 cells were plated in 24-well plates and incubated with DTP at different concentrations for 24 h followed by illumination at 16 J/cm^2^ for 17 min. Eight hours later, the cells were stained with 25 µM Hoechst 33342 (Solarbio, China) for 20 min at 37°C in the dark. The cells were washed with PBS three times and observed under a fluorescence microscope to detect changes in the nuclei.

### Western blotting

MCF-7 and SGC7901 cells were incubated with DTP for 24 h in the presence of 12 µM SB203580 or N-acetyl cysteine (NAC). PDT was performed at a light density of 16 J/cm^2^ for 17 min. Eight hours after DTP-PDT, the protein was extracted with 100 µL of radioimmunoprecipitation assay buffer (RIPA) with protease inhibitors followed by centrifugation at 12,000 rpm for 10 min at 4°C. The protein concentration was quantified using a BCA protein assay kit (Beyotime). Proteins were separated using 12% sodium dodecyl sulfate‒polyacrylamide gel electrophoresis and then transferred onto PVDF membranes. The membranes were blocked for 1 h with 5% skimmed milk in TBST (Tris buffered saline containing 0.1% Tween-20) and then incubated overnight at 4°C with the following primary antibodies: rabbit polyclonal antibody against Bcl-2 (1:1000; Abmart, USA; RRID:AB_2929011), Bax (1:1000; Cell Signaling Technology, USA; RRID:AB_10695870), P62 (1:1000; Cell Signaling Technology; RRID:AB_2798858), and LC3B (1:2000; Abmart, USA; RRID:AB_2929010), and mouse monoclonal antibodies against β-actin (1:4000; Cell Signaling Technology; RRID:AB_2223172). After washing in TBST buffer three times, the membranes were incubated with HRP-conjugated goat anti-rabbit IgG (1:4000; Cell Signaling Technology; RRID:AB_2099233) and HRP-conjugated goat anti-mouse IgG (1.5:5000; Proteintech, USA; RRID: AB_2722565) secondary antibodies for 1 h at room temperature. Proteins were visualized with an enhanced chemiluminescence detection system and semiquantified with ImageJ software.

### Preparation of cell samples

MCF-7 cells were seeded into 6-cm diameter culture dishes at a density of 1 × 10^6^ cells for 24 h. The cells were then exposed to 0.7 µM DTP for 24 h followed by illumination at 16 J/cm^2^ for 17 min. Eight hours later, the total RNA was extracted. The experiment was divided into two groups: the control and DTP-PDT groups. There are three samples for every group.

### RNA extraction and quality test

Total RNA was isolated with TRIzol reagent (Beyotime) according to the manufacturer’s instructions. Briefly, after discarding the DMEM, 1 ml of TRIzol was added to each culture dish to lyse the cells. The solution was transferred into a 1.5-mL sterile eppendorf tube. Then, 200 µL of chloroform was added to the EP tube followed by vigorous shaking for 30 s and incubation for 2–3 min at room temperature. The solution was centrifuged at 12,000 rpm for 15 min at 4°C. The supernatant was transferred into a new 1.5-mL EP tube with 500 μL isopropyl alcohol at room temperature for 10 min. The solution was centrifuged at 12,000 rpm for 10 min at 4°C. The precipitate was washed with 1 mL of 75% ethanol followed by centrifugation at 12,000 rpm for 5 min at 4°C. The supernatant was discarded, and the RNA precipitate was dissolved in 20 µL of RNase-free water. The concentration and purity of RNA were determined using a NanoDrop 2000 (Thermo Scientific, MA, USA).

### Screening of differentially expressed mRNAs

Sequencing libraries were generated and RNA sequence were analyzed with NovaSeq 6000 platform (Illumina) by genechem. HTSeq (V0.9.1, genechem, Shanghai, China) was used to analyze. Read count is positively correlated with the expression level of genes, as well as the length and sequencing depth of genes. To make the gene expression levels comparable, fragments per kilobase per million fragments (FPKM) was chosen for normalization of expression levels. FPKM is the number of fragments per thousand bases per pillion fragments of a gene. For pair-end sequencing, each fragment included two reads, and FPKM only calculated the number of fragments that matched the same transcript in the two reads. DESeq software (V1.20.0) was used for differential expression analysis between the control and DTP-PDT groups. *p* < 0.05 and |log_2_FC| > 0 were considered to be differentially expressed mRNAs (DERs). These genes were then used for further analysis.

### Gene ontology (GO) and kyoto encyclopedia of genes and genomes (KEGG) pathway enrichment analysis

Gene Ontology enrichment analysis of DERs was performed using topGO, and the *p*-value was calculated with the hypergeometric distribution method to determine the enrichment GO terms of DERs and to confirm their biological functions. The software ClusterProfiler (V3.4.4) was used for the KEGG pathway enrichment analysis of differentially expressed mRNAs.

### Disease ontology (DO) and reactome enrichment analysis

ClusterProfile software was used to perform DO and Reactome enrichment analysis of DERs.

### Protein-protein interaction (PPI) network construction

Based on the interactive relationship of proteins encoded by differentially expressed mRNAs, a protein-protein interaction network was constructed using the online database STRING (https://string-db.org/) to reveal pairwise relationships between DERs. The top 150 interacting PPI pairs with interaction scores >0.95 were screened for mapping. The cytoHubba plug-in in Cytoscape software was used to screen the top 10 interacting genes according to four different algorithms: degree, betweenness, closeness, and radiality.

### Statistical analysis

All quantitative data were analyzed using GraphPad Prism 8.0 software. Data are presented as the mean ± standard deviation (SD) of at least three independent experiments. Data were compared with one-way ANOVA for intergroup data. *p* < 0.05 was considered statistically significant.

## Results

### Inhibitory effects of DTP-PDT on MCF-7 and SGC7901 cells

The structure of DTP is shown in Fig. 1A. The absorbance spectrum of DTP exhibited five strong absorption peaks at wavelengths of 432, 522, 560, 596, and 652 nm (Fig. 1B). Because the penetration depth of light in PDT is proportional to the wavelength, 650 nm was selected as the excitation wavelength. We first assessed the phototoxicity of DTP in cancer cells. After cells were incubated with DTP for 24 h, the survival rate of MCF-7 (Fig. 1C) and SGC7901 (Fig. 1D) cells was measured using a CCK-8 assay. DTP-PDT resulted in dose- and energy-dependent growth inhibition of MCF-7 and SGC7901 cells, whereas DTP alone (without light irradiation) had no effect (Figs. 1E and 1F).

### MAPK pathway in DTP-PDT-induced inhibition of cell proliferation

**Figure 2:**
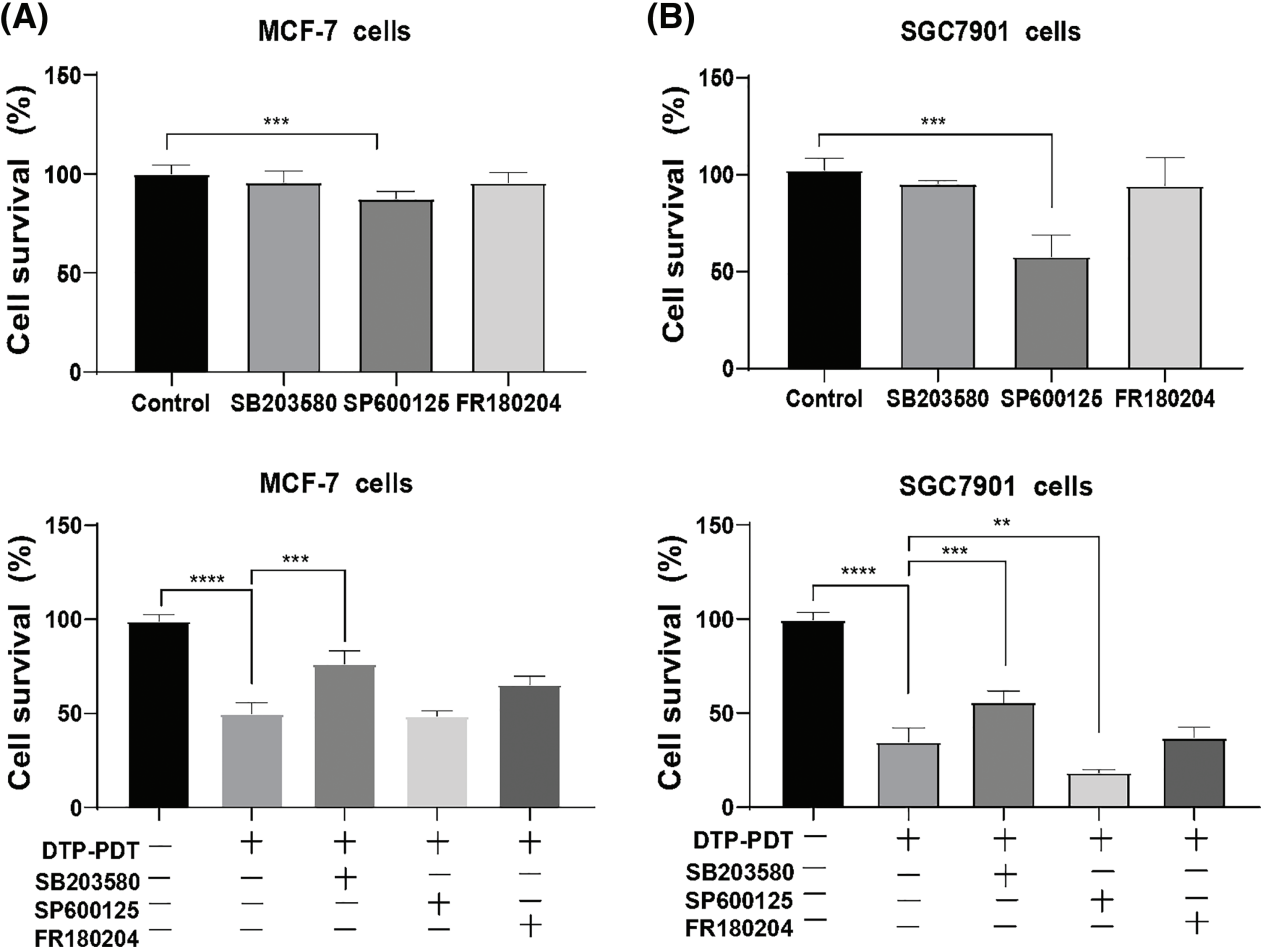
Effects of the MAPK signaling pathway on DTP-PDT. MCF-7 and SGC7901 cells were treated with 0.7 µM DTP for 24 h and then irradiated with light (16 J/cm^2^). After 8 h of irradiation, cell viability was tested using CCK-8 assay. The P38 inhibitor SB203580, JNK inhibitor SP600125, or ERK inhibitor FR180204 was used alone on (A) MCF-7 cells and SGC7901 cells. (B) SB203580, SP600125, or FR180204 combined with DTP-PDT in MCF-7 cells and SGC7901 cells. **p < 0.01; ***p < 0.001; and ****p < 0.0001.

As shown in Fig. 2A, the P38 inhibitor SB203580, JNK inhibitor SP600125, or ERK inhibitor FR180204 used alone had no significant effects on the growth of MCF-7 cells. SB203580 or FR180204 alone did not affect growth of SGC7901 cells, whereas SP600125 inhibited proliferation (Fig. 2B; *p* < 0.001).

DTP-PDT demonstrated significant inhibitory effects on MCF-7 and SGC7901 cells (*p* < 0.001; Figs. 2C and 2D) and SB203580 partly abrogated DTP-PDT-induced growth inhibition in both cell types (*p* < 0.001; Figs. 2C and 2D). FR180204 also abrogated DTP-PDT-induced growth inhibition in MCF-7 cells, but there was no significant difference in comparison with the control group. The JNK inhibitor SP600125 did not inhibit DTP-PDT-induced cytotoxicity in either MCF-7 or SGC7901 cells. Therefore, we propose that the MAPK pathway is involved in DTP-PDT’s antitumor effects.

### DTP-PDT induces ROS generation, and cytotoxicity is inhibited by NAC

**Figure 3:**
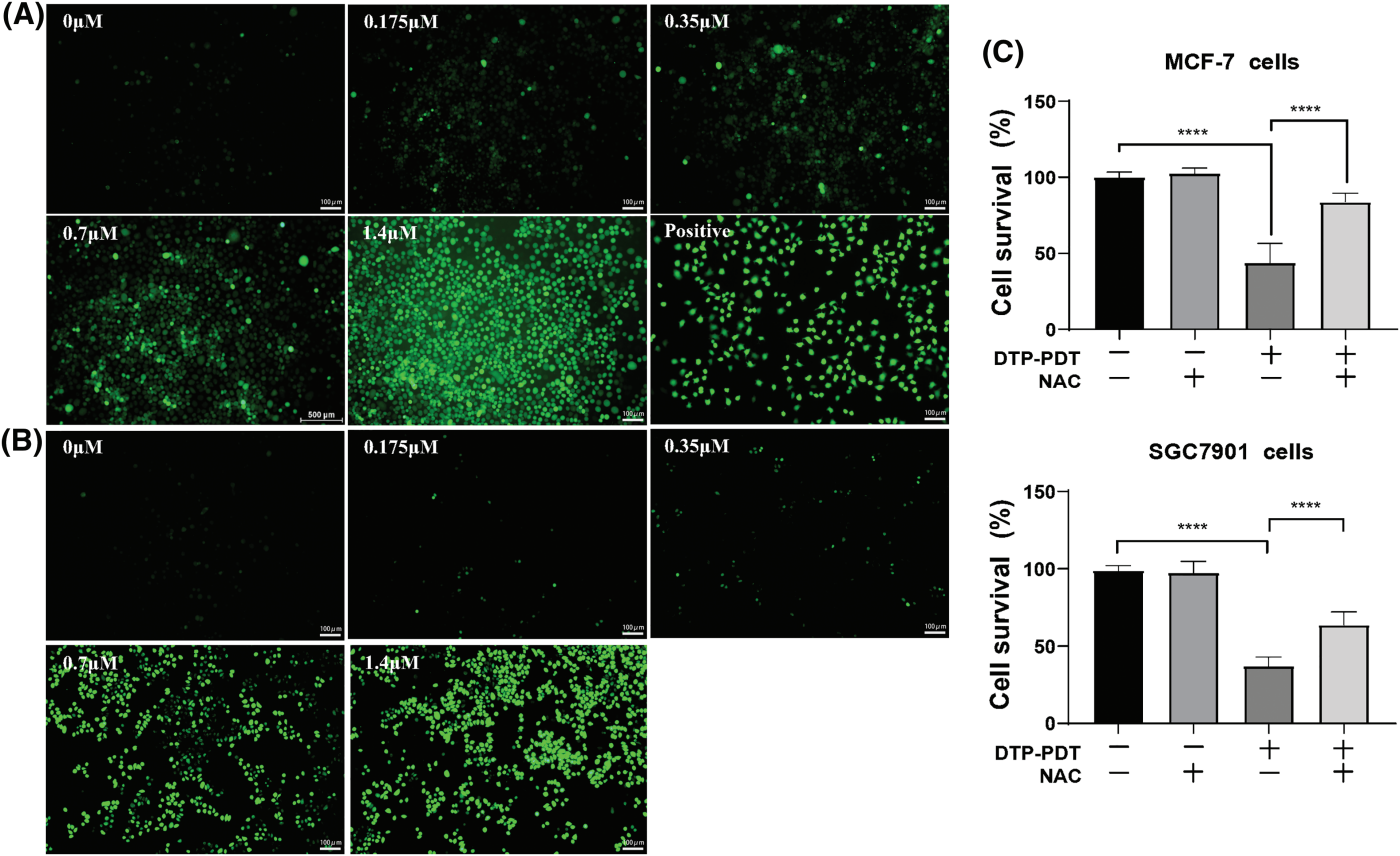
Intracellular ROS generation and the effects of the ROS inhibitor NAC on the viability of MCF-7 and SGC7901 cell lines after DTP-PDT in (A) MCF-7 and (B) SGC7901 cells. Scale bar = 100 μm. (C) The effects of NAC on the viability of cells exposed to DTP-PDT. Cell survival was measured using CCK-8 assay. ****p < 0.0001.

Reactive oxygen species are the main cytotoxic mediator of PDT and so we examined ROS generation in DTP-PDT-treated cells. In Figs. 3A and 3B, the fluorescence intensity of intracellular 2′, 7′-dichlorofluorescein (DCF) represents the production of ROS. After DTP-PDT, the fluorescence intensity of DCF in MCF-7 and SGC7901 cells increased in a dose-dependent manner, indicating an increase in intracellular ROS generation (Table 1). To confirm the role of ROS in DTP-PDT-induced cytotoxicity, the ROS scavenger NAC was used to treat cells prior to irradiation. The results indicated that DTP-PDT-induced cytotoxicity could be inhibited by blocking ROS generation with NAC (Fig. 3C).

**Table 1:** Relative fold-change of ROS within MCF-7 and SGC7901 cells compared with the control

Concentration of DTP (μM)	0.175	0.35	0.7	1.4
Relative fold-change of ROS (MCF-7 cells)	1.8	2.4	4.8	15.2
Relative fold-change of ROS (SGC7901 cells)	4.2	4.5	13.2	17.2

### DTP-PDT induces apoptosis of MCF-7 and SGC7901 cells

**Figure 4:**
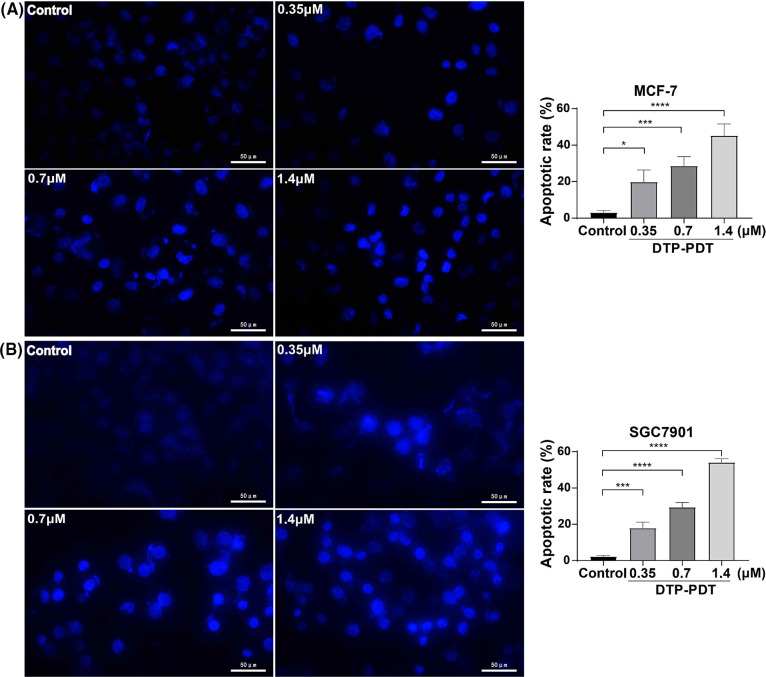
Analysis of apoptosis induced by DTP-PDT in MCF-7 and SGC7901 cells. DTP-PDT-induced morphological changes were evaluated with fluorescence microscopy and Hoechst 33342 staining in MCF-7 (A) and SGC7901 (B) cells. *p < 0.05 *vs*. the control group; ***p < 0.001 *vs*. the control group; and ****p < 0.0001 *vs*. the control group. Scale bar = 50 μm.

**Figure 5:**
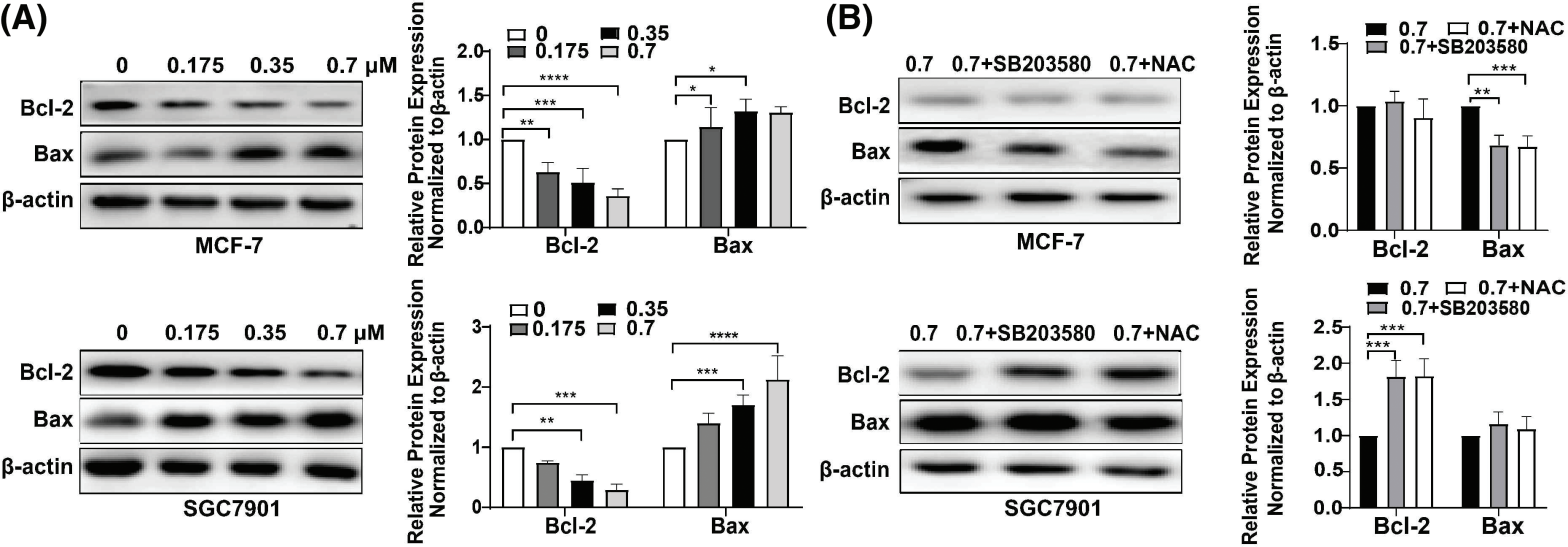
Analysis of apoptosis induced by DTP-PDT in MCF-7 and SGC7901 cells and the effects of the ROS inhibitor NAC and the P38 inhibitor SB203580. (A) Expression of apoptosis-related proteins in MCF-7 and SGC7901 cells following DTP-PDT. *p < 0.05 *vs*. the control group; **p < 0.01 *vs*. the control group; ***p < 0.001 *vs*. the control group; and ****p < 0.0001 *vs*. the control group. (B) Effects of the ROS inhibitor NAC and the P38 inhibitor SP600125 on DTP-PDT-induced cell apoptosis. **p < 0.01 *vs*. the control group; ***p < 0.001 *vs*. the control group.

To investigate the mechanism of DTP-PDT-induced cytotoxicity, the level of apoptosis was measured. After treatment with DTP-PDT, MCF-7 and SGC7901 cells demonstrated a higher density of nuclear chromatin and apoptotic bodies (Fig. 4). In the control groups, the level of apoptosis was 3.04% in MCF-7 cells and 2.21% in SGC7901 cells. Apoptosis was increased by treatment with 1.4 μM DTP-PDT to 45.23% in MCF-7 cells (*p* < 0.0001) and 54.09% in SGC7901 cells (*p* < 0.0001). The apoptotic rate was proportional to the DTP concentration. To further explore the mechanism of apoptosis, the level of mitochondrial apoptosis-associated proteins was measured. Western blot analysis demonstrated that DTP-PDT significantly elevated the expression of the proapoptotic protein Bax in MCF-7 and SGC7901 cells, whereas the level of the antiapoptotic protein Bcl-2 was decreased (Fig. 5A). Moreover, DTP-PDT-induced expression of Bax and inhibition of Bcl-2 could be reversed by the ROS inhibitor NAC or the P38 MAPK pathway inhibitor SB203580 (Fig. 5B).

### DTP-PDT induces cell autophagy in MCF-7 and SGC7901 cells

**Figure 6:**
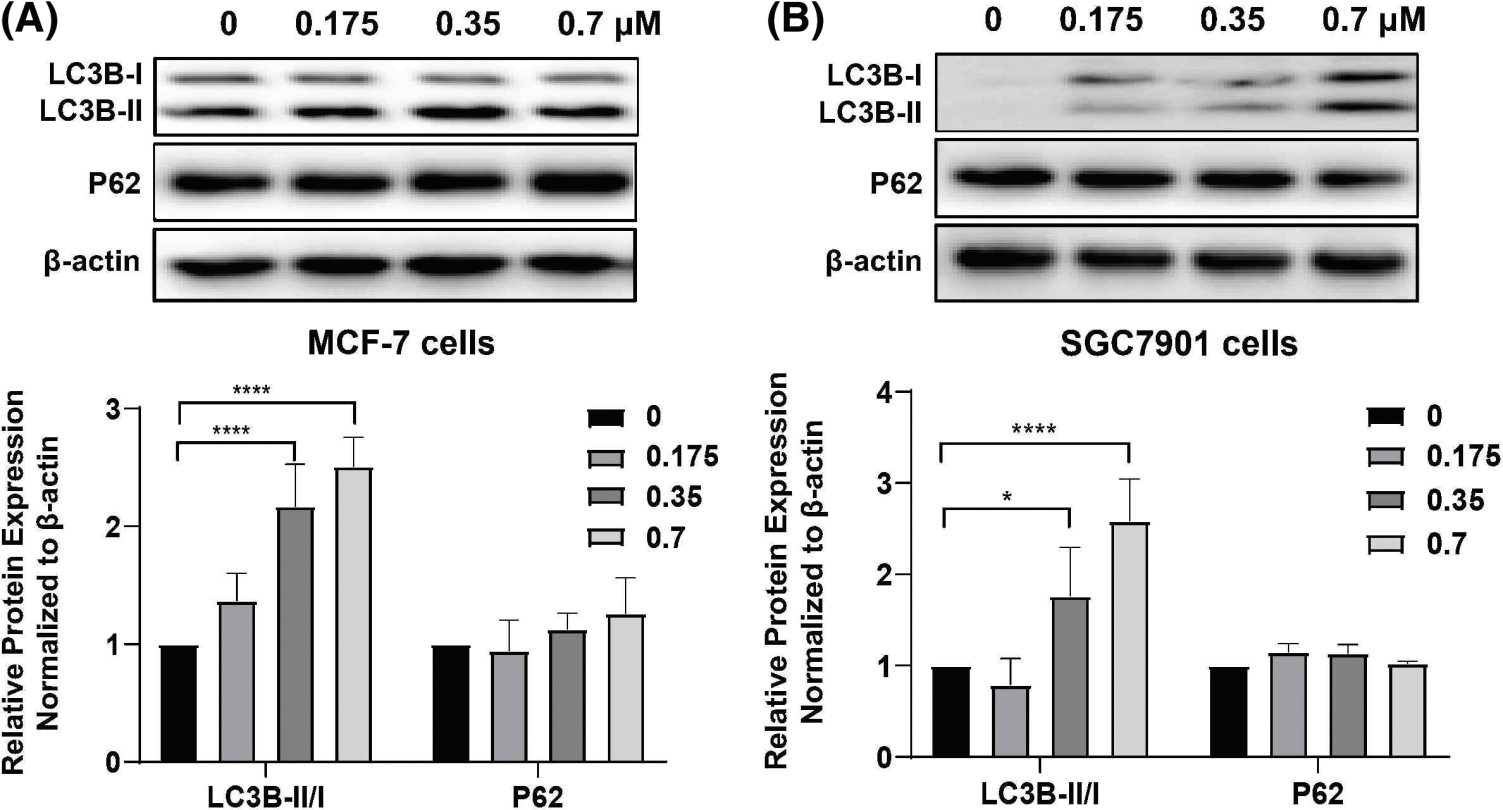
Expression of autophagy-related proteins in (A) MCF-7 and (B) SGC7901 cells following DTP-PDT. *p < 0.05 *vs*. the control group; ****p < 0.0001 *vs*. the control group.

We analyzed the changes in the autophagy-specific proteins LC3B and P62 after DTP-PDT. The ratio of LC3B-II to LC3B-I was significantly increased in a concentration-dependent manner after DTP-PDT (Figs. 6A and 6B). At a concentration of 0.7 μM, the ratios were approximately 2.5 and 2.25 in MCF-7 and SGC7901 cells compared to 0 μM group, respectively (both *p* < 0.0001). However, there was no change in the level of P62 expression in either cell line. These results indicate that cells treated with DTP-PDT undergo autophagy.

### Differential mRNA expression profile analysis in DTP-PDT-treated MCF-7 cells

**Figure 7:**
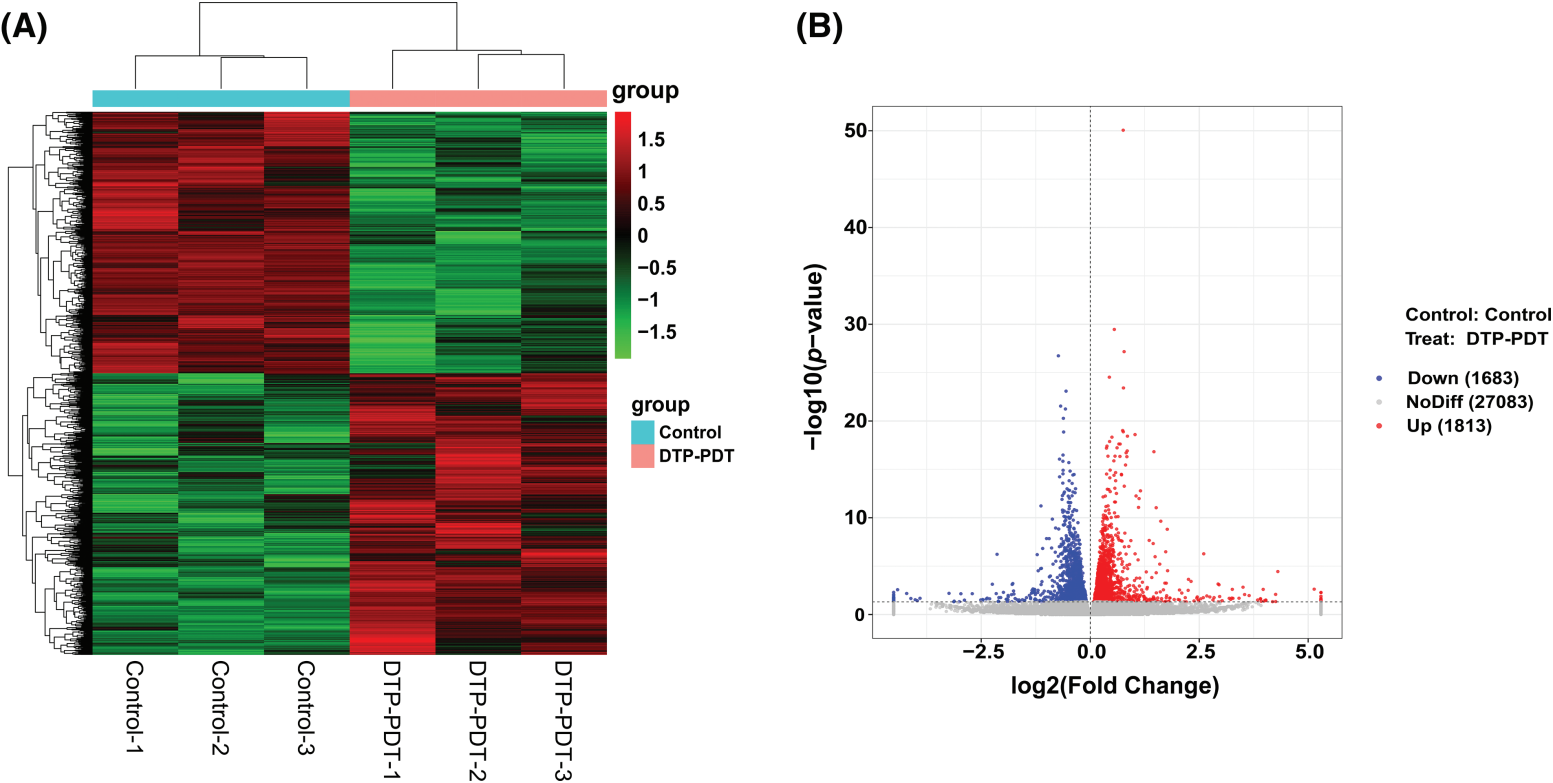
Hierarchical clustering and volcano map of differentially expressed mRNAs between the control and DTP-PDT groups. (A) Hierarchical clustering. Each column represents a cell sample and each row represents an mRNA. Red indicates an upregulated mRNA. The deeper the red color, the greater the expression level of the mRNA. Green indicates a downregulated mRNA. The lighter the green color, the greater the expression level of the gene. (B) Volcano map. The abscissa is the fold difference; the larger the absolute value, the larger the fold difference. The ordinate is the −log10 transformation of the *p*-value, and larger absolute values represent more significant differences. The red dots represent an upregulated mRNA, and the blue dots represent a downregulated mRNA.

To explore the effects of DTP-PDT on global gene expression in MCF-7 cells, RNA-seq analysis was used. A total of 3496 mRNAs were differentially expressed between the control and DTP-PDT groups (1813 upregulated by treatment and 1683 downregulated; Table 2). Hierarchical clustering of aberrantly expressed mRNAs is shown in Fig. 7A. The fold-change of DERs and the distribution of upregulated and downregulated genes are shown in a volcano map (Fig. 7B). The lists of the top 10 upregulated (Table 3) and downregulated (Table 4) mRNAs are provide according to the value of |log_2_FC|.

**Table 2:** Analysis of mRNA expression difference after DTP-PDT (|log_2_FC| > 0, *p* < 0.05)

Treat	Control	Up-regulated	Down-regulated	Total
DTP-PDT	Control	1813	1683	3496

**Table 3:** Top 10 differentially upregulatedss mRNAs arranged in order of |log_2_FC| values

mRNA	|log_2_FC|	*p*-value
*arc*	5.293	0.005
*fam153a*	4.028	0.037
*snora80d*	4.027	0.031
*rgma*	3.968	0.002
*sncaip*	3.923	0.043
*y_rna*	3.841	0.031
*elovl3*	3.645	0.011
*fcgria*	3.541	0.008
*bicdl2*	3.505	0.001
*sim2*	3.260	0.002

**Table 4:** Top 10 differentially downregulated mRNAs arranged in order of |log_2_FC| values

Gene symbol of mRNA	|log_2_FC|	*p*-value	
*rnu6-821p*	4.508	0.005	
*has1*	4.014	0.029	
*rnu6-1042p*	3.967	0.037	
*cxcl11*	3.239	0.006	
*or2w6p*	3.127	0.048	
*fcgr2a*	3.121	0.041	
*dutp6*	3.002	0.047	
*xaf1*	2.967	0.007	
*meiosin*	2.885	0.032	
*trim22*	2.501	0.031	

### GO enrichment analysis

**Figure 8:**
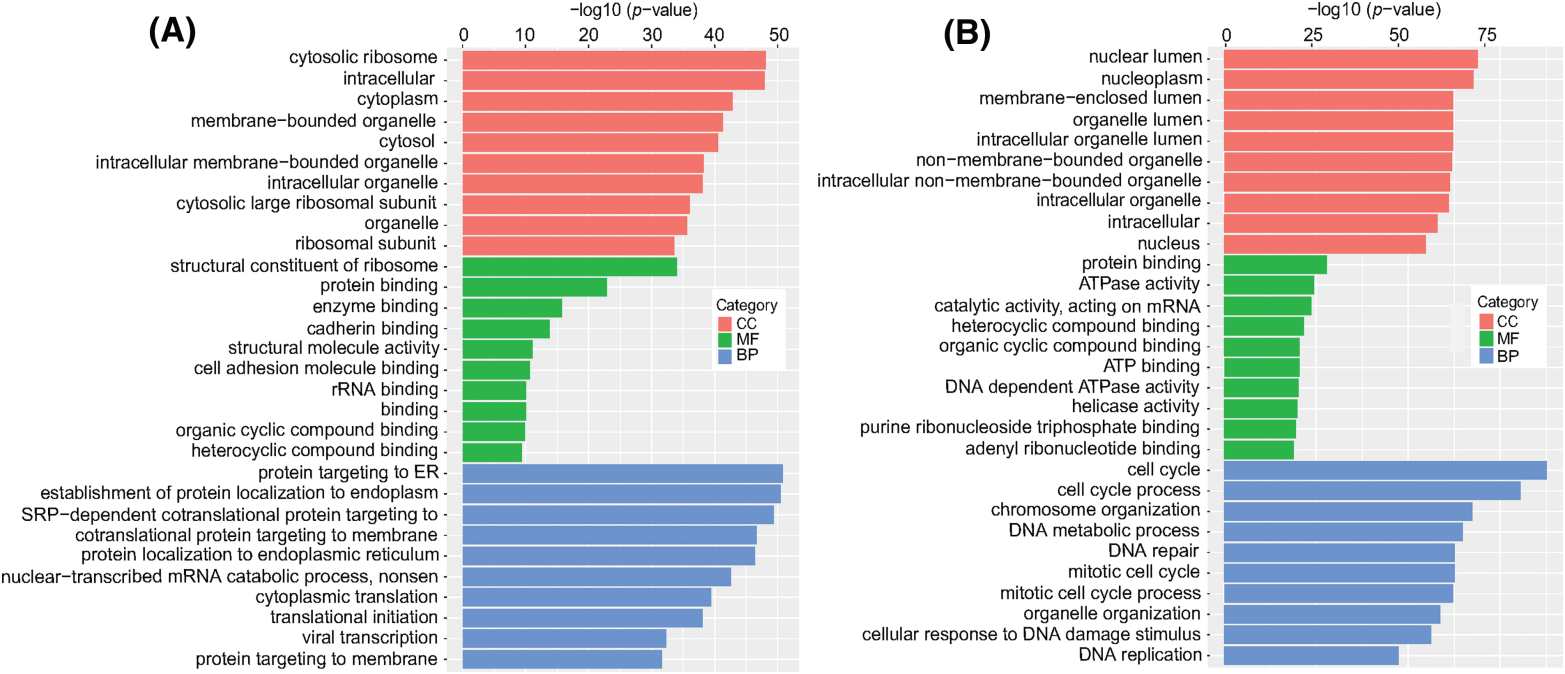
GO enrichment analysis of differentially expressed mRNAs in MCF-7 cells exposed to DTP-PDT. (A) Upregulated differentially expressed mRNAs, and (B) downregulated differentially expressed mRNAs. The ordinate is the term, and the abscissa is the −log10 (*p-*value) of enrichment for each term. *p* < 0.05 was considered statistically significant. CC represents cell component; MF represents molecular function; and BP represents biological process.

To investigate the functions of differentially expressed mRNAs between the DTP-PDT and control groups, GO and KEGG enrichment analyses were performed. The top 10 results for each enrichment with the smallest *p-*value are shown in Fig. 8. At the cell component (CC) level, the GO function of DERs was mainly enriched in the cytosolic ribosome, nuclear lumen, nucleoplasm, cytoplasm, and membrane-bounded organelle. At the molecular function (MF) level, DERs were mainly associated with ribosome structure, protein binding, ATPase activity, and enzyme binding. At the biological process (BP) level, DERs were mainly involved in the cell cycle, protein targeting to the endoplasmic reticulum, chromosome organization, and DNA metabolism.

### KEGG pathway enrichment analysis

**Figure 9:**
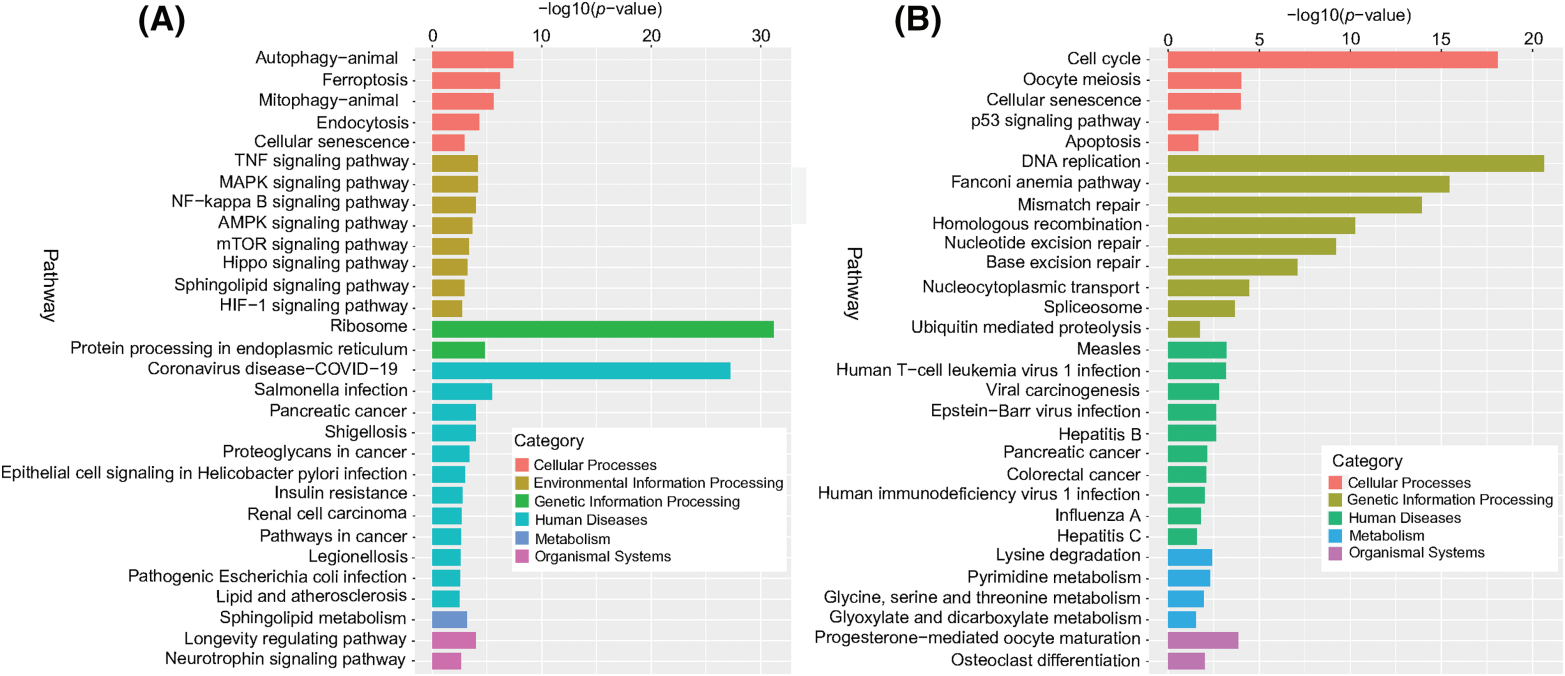
KEGG enrichment analysis of differentially expressed mRNAs. (A) Upregulated differentially expressed mRNAs, and (B) downregulated differentially expressed mRNAs. The ordinate is the pathway, and the abscissa is the −log10 (*p-*value) of enrichment for each pathway. *p* < 0.05 was considered statistically significant.

The results in Fig. 9 show that at the cellular process level, DERs were enriched in the KEGG pathways of the cell cycle, autophagy, ferroptosis, and apoptosis. At the environmental information processing level, significantly changed KEGG pathways include the TNF, MAPK, and NF-kappa B signaling pathways. At the genetic information processing level, differentially expressed mRNAs were associated with ribosomes, DNA replication, Fanconi anemia, and mismatch repair pathways. At the human disease processing level, differentially expressed mRNAs were mainly associated with coronavirus disease COVID-19, Salmonella infection, and human T-cell leukemia virus 1 infection. At the metabolism level, DERs were mainly enriched in lysine degradation, sphingolipid metabolism, and pyrimidine metabolism. At the organismal systems level, DERs were mainly involved in progesterone-mediated oocyte maturation and longevity regulation pathways.

### DO and Reactome enrichment analysis

**Figure 10:**
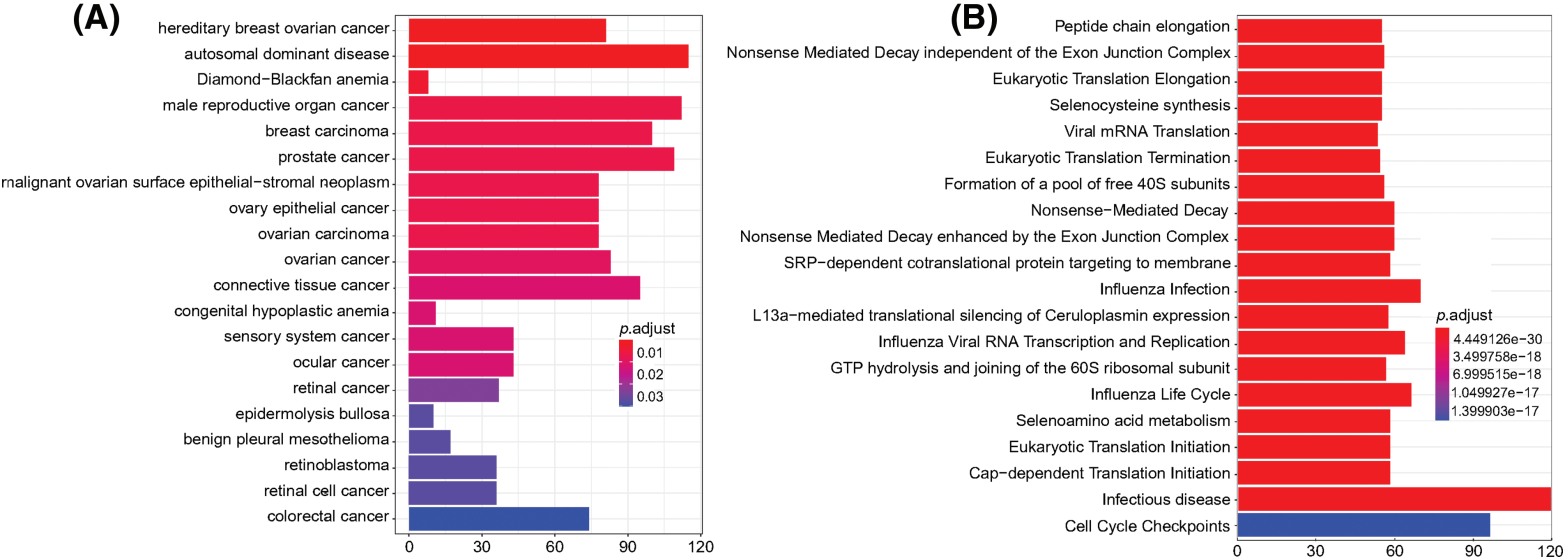
DO and Reactome enrichment analysis of differentially expressed mRNAs. (A) DO enrichment analysis. The column color represents the adjusted *p*-value, and the column height represents the number of differentially expressed genes enriched in each function. (B) Reactome enrichment analysis. The column color represents the adjusted *p*-value, and the column height represents the number of differentially expressed genes enriched in each function. *p* < 0.05 was considered statistically significant.

The DO database describes the function of human genes related to disease. The Reactome database gathers biological reactions and pathways in model species, including humans. For the DO and Reactome enrichment analysis of ERs, the top 20 results with the smallest *p-*value are shown in Fig. 10A. DERs were mainly enriched in a variety of cancers (including male reproductive organ cancer, breast cancer, prostate cancer, connective tissue cancer, ovarian cancer, and colorectal cancer) and autosomal dominant disease. The Reactome enrichment results in Fig. 10B demonstrate that DERs were mainly enriched in infectious diseases and cell cycle checkpoints.

### Protein-protein interaction networks

**Figure 11:**
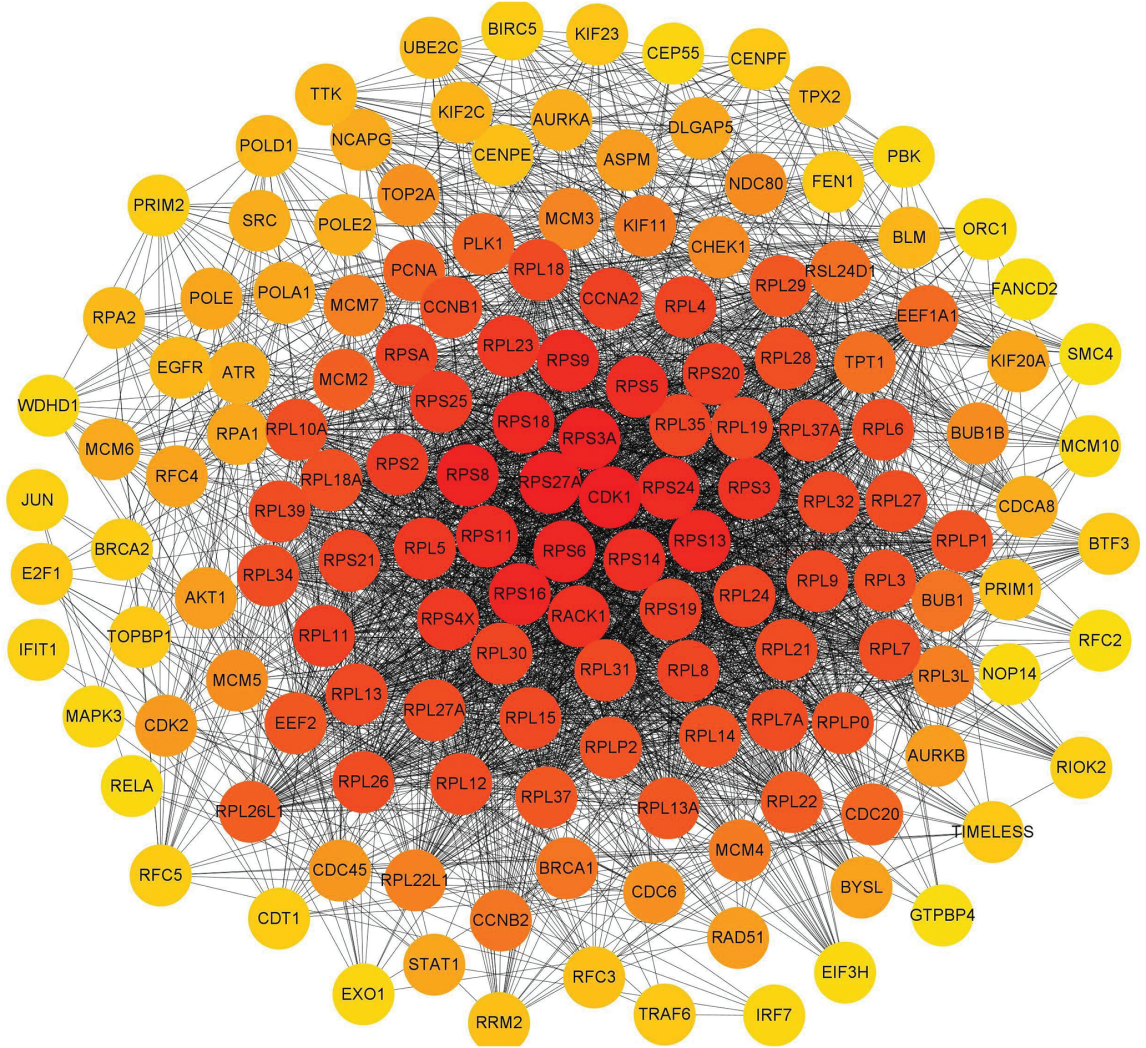
Interaction network of proteins encoded by the top 150 interacting differentially expressed mRNAs according to the degree score. The circle represents the protein, which is represented by its gene symbol; the color of the circle represents the degree score. The redder the color, the more important or the higher the score of the protein.

To investigate the interactions of DERs, a protein-protein interaction network was constructed based on the interaction of proteins encoded by differentially expressed mRNAs (Fig. 11). Each protein in the PPI network is represented by the gene name of the mRNA. The possible key connected genes were screened using the cytoHubba plug-in of Cytoscape software, and the top 10 essential hub genes were identified based on four different algorithms, including degree, betweenness, closeness, and radiality (Table 5). According to the four algorithms, *cdk1* and *rps27a* ranked in the top 10 interacting genes and may have important functions in the interaction network. According to the degree score, the top 150 interacting proteins were mapped for the PPI network (Fig. 11).

**Table 5:** Top 10 hub mRNAs screening based on 4 different algorithms in the protein–protein interaction network

Algorithms	Degree	Betweenness	Closeness	Radiality
Gene symbol of mRNA	*cdk1*	*akt1*	*cdk1*	*akt1*
	*rps27a*	*src*	*rps27a*	*src*
	*rps3a*	*rps27a*	*src*	*cdk1*
	*rps8*	*cdk1*	*akt1*	*rps27a*
	*rps6*	*egfr*	*pcna*	*brca1*
	*rps16*	*brca1*	*brca1*	*cdknia*
	*rps13*	*hspa5*	*rack1*	*pcna*
	*rps18*	*pcna*	*ccnb1*	*traf6*
	*rps9*	*traf6*	*ccna2*	*hifia*
	*rps11*	*rack1*	*cdknia*	*egfr*

## Discussion

Photodynamic therapy uses a light-sensitive material as a photosensitizer that has a higher affinity for tumors than for normal tissue. By exposure to laser irradiation, the photosensitizer in tumor tissue can generate singlet oxygen and free radicals to damage cells, eventually interfering with the growth of tumor cells and leading to cell death [13,14]. PDT has been shown to be effective in the clinic and to have benefits including a less invasive therapeutic procedure, negligible systemic toxic effects, and a lack of intrinsic or acquired resistance mechanisms. With several recent technological improvements, PDT has the potential to become an important antitumor treatment. Currently, 5-aminolevulinic acid (ALA), Photofrin, and Temoporfin are the most common clinically used photosensitizers, and the excitation wavelengths are 630, 635, and 652 nm, respectively [15]. According to their wavelengths, the tissue penetration depths for these three drugs are 5, 5, and 10 mm. PDT has been studied in skin tumors, head and neck tumors, digestive system tumors, intraperitoneal malignancies, urinary system tumors, non-small cell lung cancer and mesothelioma, and brain tumors. Although PDT is a new and promising antitumor strategy, its full potential is yet to be understood, and its range of applications alone or in combination with other approved or experimental therapeutic approaches has not been clarified. Additionally, its mechanisms are not fully understood [16–18]. In this study, the photosensitizer DTP with an absorption wavelength of 650 nm showed inhibitory effects on MCF-7 and SGC7901 cells at doses of 0.35–1.4 μM *in vitro*, which is lower than the mM-level required for 5-ALA [19].

We evaluated the effects of DTP-PDT on human breast cancer MCF-7 cells and human gastric cancer SGC7901 cells. The cell viability was significantly inhibited in a concentration-dependent manner. No cytotoxicity was seen with DTP treatment alone and when no irradiation was used. This indicates that DTP-PDT-mediated cytotoxicity in tumor cells was caused by DTP after irradiation.

PDT could kill cancer cells via various cell death pathways including apoptosis, necrosis and autophagy [20,21]. Present study found that DTP-PDT significantly induced cell apoptosis with morphological changes. The process of apoptosis can be initiated via two major pathways: extrinsic (or death receptor) and intrinsic (or mitochondrial) [22]. The latter is triggered by Bcl-2 family proteins (including proapoptotic Bax protein and antiapoptotic Bcl-2 protein) followed by the release of cytochrome C from the mitochondria [22]. In the present study, western blotting demonstrated an increase in the ratio of Bax to Bcl-2, which indicates that DTP-PDT induces tumor cell death via the intrinsic apoptotic pathway. This result is consistent with Chiu’s report [23] that Pc4-mediated PDT induces Bax translocation from the cytosol to the mitochondria and promotes activation of the intrinsic apoptotic pathway in MCF-7 cells. Temoporfin is the active pharmaceutical ingredient in the medicinal product Foscan® which is on the market in the EU for the palliative treatment of head and neck cancer [24]. Temoporfin is a second-generation porphyrin photosensitizer whose excitation wavelength is also 650 nm, which is close to the excitation wavelength of DTP. It was found that temoporfin mediated photodynamic therapy could induce cell apoptosis but no necrosis [25].

Autophagy is a process during which self-damaged organelles and proteins separate in autophagic vacuoles and are transported to lysosomes for catabolism [26,27]. Our study shows that DTP-PDT induces autophagy in MCF-7 and SGC7901 cells. The expression level of LC3B-II increased greatly with the concentration increase of DTP-PDT.

The main mechanism of cell death induced by PDT is via ROS [28]. Here, we observed that the level of ROS in MCF-7 and SGC7901 cells significantly increased after DTP-PDT and that ROS scavenger NAC could partly abrogate DTP-PDT-induced cytotoxicity. We show that DTP-PDT induced excessive ROS generation in MCF-7 and SGC7901 cells and resulted in cell growth inhibition.

The MAPK signal pathway proteins are a group of serine/threonine kinases that can be activated by various signals and transfer cell surface signals to the nucleus and cause biological reactions, such as cell proliferation, differentiation, and apoptosis [29]. Five parallel MAPK signaling pathways exist in mammals: extracellular regulated protein kinase (ERK), c-Jun N-terminal kinase (JNK), P38, extracellular signal-regulated kinases 3/4 (ERK3/ERK4), and ERK5 [30]. Different extracellular stimuli can activate different pathways. The P38 MAPK pathway can be activated by ultraviolet irradiation, stress, and inflammatory factors [30]. We investigated the involvement of the MAPK signaling pathways in DTP-PDT-mediated cytotoxicity. Because the inhibition of the P38 MAPK pathway by SB203580 could partially reverse DTP-PDT-induced cell death and decrease the levels of Bax or increase the level of Bcl-2 in MCF-7 and SGC7901 cells after DTP-PDT, we propose that the P38 MAPK pathway is involved in DTP-PDT-mediated cytotoxicity. Moreover, there was significant enrichment of DERs in the MAPK signaling pathway, which supports our above findings. Therefore, we speculate that the activation of the P38 MAPK signaling pathway is involved in DTP-PDT-mediated antitumor effects. MAPKs can be triggered by a variety of oxidant stresses, including ROS, and have been reported to contribute to the cellular responses to PDT [31,32]. Therefore, the P38 MAPK pathway might be the downstream signal of ROS.

In addition, the mechanisms of DTP-PDT through ROS could not completely explain the effect of DTP-PDT on cell viability. Just what we have talked previously, the protective effects of NAC were incomplete and NAC only partly reversed DTP-PDT-induced cytotoxicity, which suggested that other mechanisms may be involved in DTP mediated cell damage. So, we adopted the transcriptome experiment combined with bioinformatic analysis to clarify the other mechanisms. Bioinformatic analysis of DERs is a technique to identify the differences in gene expression at the transcriptional level [33]. It is an effective approach to reveal the mechanism of a drug. In the biological process category, slight changes in one or some genes in a pathway may cause changes in the function of the entire pathway, which is far more important than large changes in a single gene. Gene enrichment analysis focuses on the consistency of gene expression in gene sets with specific functions, rather than certain genes that are significantly altered, and can analyze the changes in gene expression at the global level [34]. To further explore the mechanisms involved in DTP-PDT, we analyzed the differentially expressed genes between the control and DTP-PDT groups at the transcriptional level in MCF-7 cells. There was a significant difference in mRNA expression between the control and DTP-PDT groups, and the biological functions and related pathways of DERs were associated with the cell cycle, apoptosis, autophagy, DNA replication, ribosomes, protein binding, and the MAPK pathway, suggesting that DTP-PDT-mediated cell damage was associated with these processes. Of these, apoptosis, autophagy, and the MAPK pathway are consistent with our earlier findings. We also analyzed the genome disease-related database and found that some DERs were closely related to human tumors and infectious diseases. Among them, enrichment in infectious disease was consistent with the results of KEGG enrichment analysis.

According to the interaction of DERs in the PPI network, we identified the top 10 interacting key genes. Among them, the cyclin-dependent kinase 1 (*cdk1*) and ribosomal protein 27A (*rps27a*) genes were the most important and may play a role in DTP-PDT-mediated cytotoxicity in MCF-7 cells. The protein encoded by the *cdk1* gene belongs to the serine/threonine kinase family of proteins and is essential for driving every phase of the cell cycle [35]. The protein encoded by the *rps27a* gene belongs to the ribosomal protein S27AE family and is an RNA-binding protein that performs extraribosomal functions, including ribosome biosynthesis, and posttranslational modification [36]. These results are consistent with the results of GO and KEGG enrichment analyses, which indicated that the function of differentially expressed mRNAs was associated with the cell cycle, DNA replication, ribosomes, and protein binding. Furthermore, in the present study, the *cdk1* gene was downregulated, and the *rps27a* gene was upregulated in MCF-7 cells. Therefore, DTP-PDT might inhibit cell proliferation by upregulating the expression of some ribosomal proteins and inhibiting CDK1 protein to cause cell cycle arrest.

In conclusion, our present study indicates that DTP-PDT induces apoptosis and autophagy in MCF-7 and SGC7901 cells. DTP-PDT-induced ROS production and activation of the P38 MAPK signaling pathway were involved in DTP-PDT-mediated antitumor effects. DTP-PDT had an influence on gene transcription in MCF-7 cells, especially genes related to cell cycle processes, ribosomes, DNA replication, and protein binding. *cdk1* and *rps27a* may be the hub genes involved in DTP-PDT (Fig. 11). Further research is needed to explore the precise role of these genes in DTP-PDT of cancer to reveal the mechanism of DTP-PDT. The present study is important for further in-depth research on the mechanism of DTP-PDT in the treatment of tumors.

## Data Availability

The raw data supporting the conclusions of this article will be made available by the authors.
